# Fréquence et facteurs de risque maternels de la mort fœtale in utero à Kamina, République Démocratique du Congo

**DOI:** 10.11604/pamj.2016.23.114.7817

**Published:** 2016-03-17

**Authors:** Ignace Bwana Kangulu, Albert Mwembo Tambwe A'Nkoy, John Ngoy Lumbule, Elie Kilolo Ngoy Umba, Michel Kabamba Nzaji, Prosper Kalenga Muenze Kayamba

**Affiliations:** 1 Faculté de Médecine, Département de Gynécologie et Obstétrique, Université de Kamina, République Démocratique du Congo; 2 Faculté de Médecine, Département de Gynécologie et Obstétrique, Ecole de Santé Publique, Université de Lubumbashi, République Démocratique du Congo; 3 Faculté de Médecine, Département de Santé Publique, Université de Kamina, République Démocratique du Congo

**Keywords:** Epidémiologie, mort fœtale in utero, Kamina, Epidemiology, fetal intrauterine death, Kamina

## Abstract

**Introduction:**

La mort fœtale in utero constitue un problème fréquent dans la pratique obstétricale. Les objectifs de cette étude étaient de déterminer la fréquence et d'identifier les facteurs de risque de la mort fœtale in utero à l'Hôpital Général de Référence de Kamina.

**Méthodes:**

L’étude était effectuée en deux temps. En premier lieu, une étude descriptive transversale sur 379 accouchements qui avait permis de déterminer la fréquence de la mort fœtale in utero. La détermination des facteurs de risque était faite à l'aide d'une étude cas-témoins dans laquelle les caractéristiques de 53 morts in utero ont été comparées à 106 témoins constitués des naissances vivantes et à terme.

**Résultats:**

La fréquence de la mort fœtale in utero à l'Hôpital Général de Référence de Kamina était de 13,9%. Après ajustement, l’âge maternel de plus de 35 ans (OR = 6,23; IC= (1,30-29,80)), l'antécédent de mort fœtale in utero (OR = 3,13; IC= (1,06-9,27)) et la maladie au cours de la grossesse (OR = 31,6, IC= (7,66-130,71)) ont été retenus comme facteurs significativement associés à l'augmentation de la survenue de la mort fœtale. L'instruction élevée de la mère (OR = 0,11; IC= IC= [0,03-0,42]) et la résidence à Kamina (OR = 0,23; IC= (0,08-0,62)) diminuaient ce risque.

**Conclusion:**

La fréquence de la mort fœtale in utero était de 13,9%. L’âge maternel avancé l'antécédent de mort in utero et la maladie au cours de la grossesse étaient associés à la mort fœtale in utero mais par contre, l'instruction élevée de la femme et la résidence à Kamina diminuaient le risque. La surveillance des gestantes à risque, le dépistage et la prise en charge des maladies pendant la grossesse s'avèrent nécessaires dans la perspective de réduire la fréquence de la mort fœtale in utero dans notre milieu.

## Abstract (en)

**Introduction:**

Fetal intrauterine death constitutes a frequent problem in obstetric practice. The objectives of this study were to determine the frequency and to identify risk factors of fetal death at Reference General Hospital of Kamina.

**Methods:**

The study was done in two times. First, a cross-sectional descriptive study of 379 deliveries which allowed to determine the frequency of fetal death. Then, the determination of risk factors was made using a case-control study in which the characteristics of 53 fetal deaths were compared to 106 controls constituted of live births.

**Results:**

The fetal intrauterine death's frequency at Reference General Hospital of Kamina was at 13,98%. After adjustment, maternal age over 35 years (OR = 6,23; IC= (1,30-29,80)), antecedent of fetal intrauterine death (OR = 3,13; IC= (1,06-9,27)) and diseases during pregnancy (OR = 31,6, IC= [7,66-130,71]) have been identified as factors significantly associated with increased occurrence fetal death. The high maternal education (OR = 0,11; IC= (0,03-0,42)) and Kamina's residency (OR = 0,23; IC= (0,08-0,62)) decreased this risk.

**Conclusion:**

The frequency of fetal death was 13,9%. The advanced maternal age, the history of fetal death and disease during pregnancy was associated with fetal intra uterin death but the high maternal instruction and the Kamina residence reduced the risk. The monitoring of at-risk pregnant, screening and the treatment of diseases during pregnancy are necessary in view of reducing prevalence of fetal death in our midst.

## Introduction

La santé de la mère, du nouveau-né et de l'enfant figure parmi les droits fondamentaux des femmes et des enfants [1–3]. Depuis les sommets mondiaux de la femme et de l'enfant, cette problématique a pris de plus en plus place dans les débats publics et la santé du couple mère-enfant a été ainsi inscrite dans les divers agendas internationaux [1, 2]. Des millions de mortinaissances non comptabilisées et non reflétées dans les politiques mondiales surviennent chaque année. Jusqu’à maintenant, les systèmes de collecte de données des Nations Unies ne comptent pas les mortinaissances qui, pourtant, constituent une réalité quotidienne dans le monde entier. Les objectifs politiques mondiaux, comme les Objectifs du Millénaire pour le Développement(OMD), ne prennent pas suffisamment compte des mortinaissances, tout comme la charge mondiale de morbidité. Un tel silence cacherait la gravité du problème et empêcherait l'investissement. Pourtant, la connaissance du nombre de mortinaissances, de leurs causes ainsi que des solutions éventuelles sont la clé pour concevoir des politiques et des programmes efficaces [4]. La mort fœtale in utero (MFIU), comprise dans la mortinaissance, se définit par tout décès fœtal avant la mise en travail, survenant après la limite de la viabilité fœtale telle qu'elle a été fixée par l'OMS, à savoir 22 semaines d'aménorrhées (SA) ou un poids de naissance de plus de 500 grammes [5]. Elle est l'une des complications majeures de la grossesse dont on puisse être témoin en tant que praticien. C'est un évènement traumatisant et à la base de nombreuses questions non seulement pour la femme enceinte mais aussi pour l'obstétricien. Sa prévalence varie à travers le monde d'une région à une autre: avec 2% dans le monde et 0,5% dans les pays à hauts revenus [6]. Cependant, dans notre pays, les données ne sont pas disponibles pour apprécier l'ampleur du problème, ce qui nous a poussé à initier la présente étude. L'objectif général de cette étude était de contribuer à l'amélioration du bien-être du couple mère-enfant par la prise en charge préventive de la mort fœtale in utero à Kamina. De cette préoccupation principale se dégagent les objectifs spécifiques ci-après: déterminer la fréquence de la mort fœtale in utéro à l'Hôpital Général de Référence de Kamina; identifier les principaux facteurs de risque maternels.

## Méthodes

**Cadre, type et période d’étude:** Il s'agitssait d'une étude descriptive transversale puis cas-témoins menée à l'Hôpital Général de Référence de Kamina (HGR/Kamina) sur une période de 16 mois, allant du 1^er^ janvier 2014 au 30 avril 2015. L’étude descriptive transversale avait permis de déterminer la fréquence tandis que l'identification des facteurs de risque était faite par l’étude cas- témoins. Les mères avec mort fœtales ont été comparées aux mères avec naissances vivantes à terme afin de dégager les différents facteurs de risque associés à cette anomalie de développement de la grossesse.

**Population d’étude et échantillon:** Cette étude concernait toutes les gestantes admises à l'HGR/Kamina pendant la période de notre recherche pour mort fœtale in utero. Ces dernières ont été comparées à un groupe de témoins issus de la même structure et de la même période. Chaque cas de mort fœtale in utero a été couplé à 2 témoins. Ainsi, 53 cas ont été appariés à 106 témoins constitués des naissances vivantes à terme.

**Critères d'inclusion et d'exclusion:** Sont inclus dans cette étude les cas de mort fœtale in utero ainsi que leurs témoins choisis selon les critères de sélection ci-dessous décrits. Dans le groupe des cas, les morts fœtales intrapartales et celles survenues avant l’âge de viabilité fœtale sont exclus. Dans celui des témoins, les accouchements prématurés et ceux intervenus dans le post-terme ne sont pas concernés.

**Définition d'un cas et d'un témoin:** Un cas est défini comme toute gestante admise pour mort fœtale in utero à l'Hôpital Général de Référence de Kamina ainsi que son nouveau-né pendant la période de notre étude sur grossesse d’âge de viabilité tel que fixé par l'OMS, soit un âge gestationnel supérieur ou égale à 22 semaines d'aménorrhée et ayant accepté de participer à la présente étude. En cas d'ignorance de la date des dernières règles par la gestante, un poids fœtal supérieur ou égale à 500g était utilisé comme critère. Sont pris comme témoins, 2 mères ayant accouché directement après les cas de mort fœtale, avec naissances vivantes et grossesse à terme. Ainsi, chaque cas de mort fœtale in utero a été comparé à 2 témoins.

**Collecte des données et analyse statistique:** La collecte des données a été faite sur base d'une fiche de collecte des données préétablie et pré-testée, soumise aux femmes concernées selon la langue de chacune. Les informations recueillies ont été saisies avec Excel 2010 et analysées sur le logiciel Statistical Package for Social Science (SPSS) version 19.

**Considérations éthiques:** Qu'il s'agisse du côté des cas ou de celui des témoins, notre étude n'avait aucun caractère contraignant. Toute information recueillie auprès des femmes concernées a été et restera confidentielle. De même, les noms des participantes resteront confidentiels et ne seront pas mentionnés dans la présentation des résultats ou associés aux résultats, de quelque manière que ce soit. Ils ne seront par ailleurs divulgués à aucune tierce personne.

## Résultats

**Fréquence de la mort fœtale in utero:** L’étude a enregistré 53 cas de mort fœtale in utero à l'Hôpital Général de Référence de Kamina sur un total de 379 accouchements. Ce qui donne une fréquence de 13,98%.

**Facteurs de risque maternels de la MFIU:** Il est étudié dans le Tableau 1, l'influence de l’âge de la mère, sa provenance et son niveau d'instruction sur la survenue de la MFIU. Dans le Tableau 2, les influences de la parité, la gestité et le dernier intervalle inter génésique sont concernées et en fin, la relation entre la MFIU et les antécédents médicaux de la mère, ceux d'avortement et de MFIU est étalée dans le Tableau 3. Du Tableau 1,il se dégage que le risque de la mort fœtale in utero augmentait avec l’âge, l'habitation au village et le bas niveau d'instruction de la mère. Chez les femmes avec mort fœtale in utero, la côte chez les femmes âgées de plus de 35 ans est de 6,8 (IC=[1,87-24,40]) comparativement à celles de moins de 20 ans. Elle est de 3,7 (IC= [1,66-8,12]) chez les femmes provenant aux villages et de 2,6 (IC= [1,22-5,30]) chez celles non instruites. Dans l'analyse duTableau 2, il est démontré qu'il existe une association significative entre la multigestité (gestité supérieure ou égale à 4) et la multiparité (parité supérieure ou égale à 4) et la survenue de la mort fœtale in utero. En effet, duTableau 2, il se dégage que chez les cas, la côte d’être multigeste (plus de 3 conceptions) est de 2,9 (IC= [1,19-6,80]) comparativement aux témoins. Elle devient de 3,2 (IC=[2,12-12,13]) chez les multipares. Au contraire, l'analyse des résultats sur la mort fœtale in utero et l'intervalle inter génésique ne donne pas d'association significative. Du Tableau 3, il se dégage que chez les cas, les côtes sont respectivement de 17,8 (IC= [1,52-17,84]); 3,1 (IC= [1,32-7,22]) et 6,1 (IC= [2/a>,67-14,05]) chez les femmes avec antécédents médicaux morbides, de mort fœtale in utero et celles ayant eu une maladie au cours de la grossesse, comparativement aux témoins. Il se dégage du Tableau 4 qu'après ajustement entre les différentes variables étudiées, l’âge maternel de plus de 35 ans (OR = 6,23, IC= [30-29,80]), l'antécédent de MFIU (OR = 3,13, IC= [1<,06-9,27]) et la maladie au cours de la grossesse augmentent significativement le risque de la MFIU. L'instruction élevée de la femme (OR = 0,11; IC = 0,03-0,42]) et la résidence à Kamina (OR = 0,23, IC= [0,08-0,62]) semblent diminuer ce risque.

**Tableau 1:** Répartition des cas selon l’âge, l’état civil, la provenance et le niveau d'instruction des mères

Age, provenance et niveau d'instruction	Cas (n = 53)	Témoins (n = 106)	OR [IC]	p-value
**Age de la mère**				
Inférieur à 20 ans	4(12,9%)	27(87,1%)	1	-
20 – 35 ans	35(35,0%)	65(65,0%)	3,6[1,18-11,22]	0,02
Supérieur à 35	14(50,0%)	14(50,0%)	6,8[1,87-24,40]	0,00
**Provenance**				
Villages	19(57,6%)	14(42,4%)	3,7[1,66-8,12]	0,00
Kamina	34(27,0%)	92(73,0%)		
**Niveau d'instruction**				
Aucun et primaire	40(40,8%)	58(59,2%)	2,6[1,22-5,30]	0,01
Secondaire et supérieur	13(21,3%)	48(78,7%)		

**Tableau 2:** Répartition des cas selon les antécédents obstétricaux (gestité, parité et le dernier intervalle inter génésique) et la survenue de la mort fœtale in utero

Gestité, parité et dernier intervalle inter génésique(I.I.G)	Cas (n = 53)	Témoins (n = 106)	OR [IC]	p-value
**Gestité**				
1	9(20,4 %)	35(79,6 %)	1	-
2 à 3	14(31,8 %)	30(68,2 %)	1,8[0,69-4,78]	0,22
Plus de 3	30(42,3 %)	41(57,3 %)	2,9[1,19-6,80]	0,02
**Parité**				
0	9(20,0 %)	36(80,0 %)	1	-
1	2(33,3 %)	4(66,7 %)	2,0[0,31-12,69]	0,45
2 à 3	12(30,0 %)	28(70,0 %)	1,7[0,63- 4,64]	0,29
Plus de 3	30(44,1 %)	38(55,9 %)	3,2[2,12-12,13]	0,01
**Dernier intervalle inter génésique (I.I.G)**				
≤ 2	32(38,6 %)	51(61,4 %)	1	
≥ 2	11(35,5 %)	20(64,5 %)	1,14[0,48-2,69]	0,76

**Tableau 3:** Répartition des cas selon les antécédents maternels et la survenue de la mort fœtale in utero

Facteurs	Cas (n = 53)	Témoins (n = 106)	OR	p-value
**Antécédents médicaux morbides**				
Présents	9(69,2%)	4(30,8%)	17,8[1,52-17,84]	0,00
Absents	44(30,1%)	102(69,9%)		
**Antécédent d'avortement**				
Oui	14(45,2%)	17(54,8%)	1,9[0,84-4,19]	0,12
Non	39(30,5%)	89(69,5%)		
**Antécédent de MFIU**				
Oui	15(55,6%)	12(44,4%)	3,1[1,32-7,22]	0,01
Non	38(28,8%)	94(71,2%)		
**Maladies au cours de la grossesse**				
Oui	22(66,7%)	11(33,3%)	6,1[2,67-14,05]	0,00
Non	31(24,6%)	95(75,4%)		

**Tableau 4:** Facteurs de risque de la mort fœtale in utero après ajustement

Facteurs de risque	OR brut [IC à 95%]	p	OR ajusté [IC à 95%]	p
Résidence : Kamina versus villages	0,27[0,12-0,60]	0,00	0,23 [0,08-0,62]	0,04
Age : supérieur à 35 ans versus inférieur à 20 ans	6,8[1,87-24,40]	0,00	6,23 [1,30-29,80]	0,02
Instruction : secondaire et supérieure versus aucune et primaire	0,39[0,19-0,82]	0,01	0,11 [0,03-0,42]	0,00
Antécédent de MFIU	3,1[1,32-7,22]	0,01	3,13[1,06-9,27]	0,04
Maladies au cours de la grossesse (oui versus non)	6,1[2,67-14,05]	0,00	31,6 [7,66-130,71]	0,00

## Discussion

**Fréquence:** Dans notre série, la fréquence de la mort fœtale in utero était de 13,9%. Cette dernière est très élevée comparativement aux fréquences trouvées dans certaines études notamment celles d'Andriamandimbison et al [7] au Madagascar, d'Andriamandimbison et al [8], de Mouquet et al [9] et de Marie [10] en France; de Yehia [11] au Mali, de Tamrakar et Chawla [12] au Népal, de Singh et al [13] en Inde et de Tchaou et al [14] au Bénin. Globalement, il est établi que la mort fœtale in utero est un événement ayant des prévalences très variables à travers le monde, avec une prévalence mondiale de 2% et de 0,5% dans les pays à haut revenu, les pays en voie de développement étant les plus touchés [6]. La fréquence élevée déterminée dans cette étude résulte notamment du faible niveau socio-économique, de la difficulté d'accès aux soins de santé de qualité et surtout du comportement de la femme enceinte du milieu rural et semi-rural vis-à-vis des consultations prénatales.

**Relation entre l’âge, provenance, niveau d'instruction de la mère et la MFIU:** Du Tableau 1, il se dégage que le risque de la mort fœtale in utero augmentait avec l’âge de la mère. Chez les cas, la côte des femmes âgées de plus de 35 ans est de 6,8 (IC=[1,87-24,40]) comparativement aux témoins. Cette constatation s'accorde avec les résultats d'autres auteurs ayant montré l'existence d'une augmentation des morts in utero et de la mortalité périnatale dans les grossesses tardives notamment dans les travaux de Belaish-Allart et al [15], d'Andriamandimbison et al [7], de Lansac et al [5] et de Roman et al [16]. Plusieurs raisons comme l'hypertension artérielle, le diabète sucré, l'obésité, le risque de placenta prævia et de malformations fœtales expliquent la prédisposition de la femme âgée à la MFIU. Les gestantes qui provenaient des villages entourant Kamina avaient 3,7 fois le risque (IC= [1,66-8,13]) de MFIU. Il est noté dans la littérature que le risque de mort fœtale est plus important pour les patientes appartenant à un milieu socio-économique défavorisé, même après ajustement sur le niveau socio-économique [5, 17]. Ces gestantes sont exposées à des travaux lourds, surtout champêtres, même pendant la grossesse, ne respectent pas souvent l'hygiène de la grossesse et sont mal suivies aux consultations prénatales ou les commencent tardivement. La femme rurale est soit perpétuellement soumise aux décisions du mari et de sa famille qui peuvent être souvent favorables aux interventions traditionnelles moins coûteuses, soit confrontée au problème d'inaccessibilité à des formations sanitaires (longue distance à parcourir, insuffisance des moyens de transport, routes en mauvais état,…) [18]. Chez les femmes non instruites, le risque d'avoir une MFIU est de 2,6 (IC= [1,66-8,13]) comparativement aux femmes instruites. Il existe un lien entre l'instruction et le recours aux services de santé. A travers l’école, l'individu entre en contact avec des valeurs et des croyances nouvelles. Ce sont ces savoirs nouveaux qui amènent l'individu à ajuster son comportement en matière de santé aux exigences de la modernité. Tout d'abord la perception de la maladie ou de la mort relève des croyances populaires en Afrique. Une femme instruite est capable de s'affranchir de ces croyances car elle est capable de mieux comprendre des informations relatives aux soins maternels, de développer aussi des nouveaux comportements en matière d'hygiène. En plus, l'instruction est un facteur important de changement de la représentation des relations familiales car le pouvoir de décision ne revient plus aux ainés sociaux mais à la mère ou au couple. En plus du rôle important que joue l’école dans le processus de changement de comportement, l'instruction augmente également le niveau de vie, donc la capacité de payer des soins de santé [18]. Une autre étude récente souligne le rôle du niveau d'instruction de la mère sur l'augmentation de la mortinaissance [17].

Relation entre la parité, gestité, intervalle inter génésique et la MFIU: En rapport avec le rang de la grossesse (gestité) et le nombre d'accouchements (parité) présentés au Tableau 2, il se dégage que chez les cas, la côte des multigestes (plus de 3 conceptions) était de 2,9 (IC= [1,19-6,80]) par rapport aux primigestes. Elle devient de 3,2 (IC= [2,12,13]) chez les multipares. La fréquence de la MFIU augmentait donc avec la gestité et la parité. Les multipares et les multigestes ont le plus souvent aussi un âge avancé et sont exposées à certaines pathologies incriminées dans les causes de la MFIU, telles le placenta prævia, l'HRP, l'HTA, le diabète sucré, l'obésité, le RCIU [19–21]. Et aussi, il est noté que généralement, les primipares utilisent beaucoup plus les formations sanitaires que les multipares. Il est probable que les premières soient plus inquiètes par rapport à leur état tandis que les multipares sont confiantes grâce à l'expérience accumulée dans le passé. L'utilisation des soins prénataux et des soins à l'accouchement diminue avec la parité. En effet, les femmes perçoivent des risques associés à la première grossesse et ont tendance à recourir plus aux services de santé maternelle. Quant aux multipares, elles éprouveraient également des problèmes de garde des enfants et ne pourraient pas se rendre dans des formations de santé [18]. Ce non recours aux services de santé surtout prénataux pourrait aussi justifier l'exposition des multigestes et multipares à des nombreuses complications de la grossesse parmi lesquelles l'on retrouve la MFIU. Si dans la littérature, les grossesses rapprochées sont connues comme à risque de complications maternelles, fœtales et néonatales (anémie maternelle, RCIU, la prématurité, morbidité néonatale, FPN, etc.) [22], dans notre étude, il a été trouvé que l'intervalle inter génésique inférieur à 2 ans n'est pas associé à la MFIU.

**Influence des antécédents médicaux, de MFIU et ceux d'avortement sur la MFIU:** La survenue de la mort fœtale in utero était liée aux antécédents médicaux morbides (OR = 17,8, IC= [1,52-17,84]) et à l'accouchement antérieur d'une mort fœtale (OR = 3,1, IC= [1,32-7,22]). Cependant, un antécédent d'avortement n'influençait pas la survenue de la mort du fœtus in utero (OR = 1,87, IC= [0,8434-4,1879]). L'analyse du tableau III indique que dans 28,3% des cas, au moins un antécédent de mort fœtale in utero a été noté parmi les patientes. Le taux de récidive de MFIU a été trouvé à des proportions comparables dans la littérature: 5,33% pour l’étude d'Andriamandimbison [7] au Madagascar et 4,5% pour celle de Nguyen en France [23]. Frias et al. [24], par contre, ont trouvé un taux de récidive de mort fœtale in utero de 25%, très élevé par rapport à celui trouvé dans la présente étude. Les femmes avec antécédent de mort fœtale sont plus exposées à donner naissance à un enfant mort in utero que les autres (OR = 3,1, IC [1,32-7,22]). Lors des grossesses suivant une MFIU, les résultats de Heinonen et al. [25] montrent une augmentation significative de la survenue d'hématome rétroplacentaire, de diabète gestationnel, d'accouchement avant 37 SA et d'enfants de poids inférieur à 2500 grammes mais les auteurs n'observent pas de récidive de MFIU. Ce dernier constat a été souligné aussi par Robson et al [26] et Frias et al [24]. Cependant, l’étude de Nguyen [23] trouve qu'il existe un groupe le plus à risque de récidive de la mort fœtale in utero: celui des patientes ayant eu une MFIU d'origine vasculaire avec une thrombophilie avec un taux de récidive de 18,5%. En revanche, elle n'a pas constaté de récidive chez les patientes pour lesquelles il n'avait pas été retrouvé d’étiologie à la mort fœtale. Soulignons cependant que la persistance des problèmes maternels chroniques expose la femme à des récidives de MFIU.

## Conclusion

Au terme de notre étude, il est trouvé que la fréquence de la mort fœtale in utero est de 13,98%. Parmi les nombreux facteurs étudiés, il se trouve que la survenue d'une mort fœtale in utero est significativement associée à l’âge de la mère avancé (plus de 35 ans), la provenance (les femmes vivant aux villages étant les plus concernées), au bas niveau d'instruction, à l'antécédent de MFIU et à la maladie au cours de la grossesse. La surveillance des gestantes à risque, le dépistage et la prise en charge des maladies pendant la grossesse s'avèrent nécessaires dans la perspective de réduire la fréquence de la mort fœtale in utero dans notre milieu.

### Etat des connaissance sur le sujet

- La fréquence de la mort fœtale in utero est variable suivant les conditions socio-économiques d'un milieu ou d'un pays
- Certaines caractéristiques maternelles pourraient influencer la survenue de la mort fœtale in utero.

### Contribution de notre étude a la connaissance

- La fréquence très élevée de la MFIU à Kamina, qui était jusque-là ignorée.
- Il existe des facteurs de confusion (tels que la parité, la gestité, l'intervalle inter génésique,…) au profit de l’âge de la mère, son niveau d'instruction, sa résidence au village, l'antécédent de mort fœtale in utero et la maladie maternelle au cours de la grossesse.
